# Cisplatin in combination with Phenethyl Isothiocyanate (PEITC), a potential new therapeutic strategy for malignant pleural mesothelioma

**DOI:** 10.18632/oncotarget.2604

**Published:** 2014-10-18

**Authors:** Iza Denis, Laurent Cellerin, Marc Gregoire, Christophe Blanquart

**Affiliations:** ^1^ Inserm, UMR892, Nantes, F-44000, France; ^2^ CNRS, UMR6299, Nantes, F-44000, France; ^3^ Université Nantes, Nantes, F-44000, France; ^4^ Service d'Oncologie Médicale Thoracique et Digestive, Hôpital Laënnec, CHU de Nantes, France

**Keywords:** malignant mesothelioma, cisplatin, isothyocyanate, reactive oxygen species, combination treatment

## Abstract

Malignant pleural mesothelioma (MPM) is a very aggressive form of cancer with a poor diagnosis and prognosis. The first line treatment for MPM is a combination of cisplatin and Pemetrexed, which displayed limited efficacy and severe side effects. The naturally occurring compound phenethyl isothiocyanate (PEITC) previously showed interesting anti-tumor properties on several cancer cell lines. We thus aim at evaluating PEITC used alone or in combination with cisplatin in order to improve MPM treatment.

Nine MPM cell lines and primary mesothelial cells (PMC), co-cultured or not with M2 macrophages present in MPM microenvironment, were used to assess PEITC and cisplatin anti-tumor properties. Compounds were used alone or in combination.

Both PEITC and cisplatin were cytotoxic on MPM cells in a dose dependent manner. We herein showed that PEITC-induced cytotoxicity was due to the generation of reactive oxygen species. Moreover, we showed that cisplatin-PEITC combination allowed the potentialization of both compounds' cytotoxic effects and prevented the emergence of resistant MPM cells. Interestingly, PMC were not sensitive to the combination. Finally, we showed that M2 macrophages did not alter the anti-tumor properties of the combination. Cisplatin-PEITC combination thus represents a promising strategy to induce a selective toxicity towards malignant cells.

## INTRODUCTION

Malignant pleural mesothelioma (MPM) is an aggressive neoplasm affecting the mesothelial surfaces of pleural and peritoneal cavities [1] mainly arising from a chronic exposure to and inhalation of asbestos [2]. Its aggressiveness and critical health care matter mainly arise from its late diagnosis and poor prognosis (less than a year after diagnosis). The first line treatment for MPM lies in the combination of cisplatin with an anti-metabolite: the Pemetrexed (Alimta). There are several clinical trials relating this combination to treat MPM [3-4], that lead to better response compared to cisplatin alone [5]. However, this improvement remains modest and only half of the patients respond to the combination [6]. It is thus necessary to find new therapeutic approaches to treat MPM.

It has now been described for many years that frequent consumption of cruciferous vegetables reduces the incidence of cancer [7]. The active compounds primarily responsible for those cancer chemopreventive properties were described to be the glucosinolates [8-9], which are substituted B-thioglucoside N-hydroxysulfates synthesized by the plant from 8 amino acids. However, the inhibition of carcinogenesis seems to rather be attributable to some of their breakdown products: the isothiocyanates (ITCs) [10].

ITCs anticarcinogenic properties mechanisms of action are still under investigation but it is now established that it stems from their ability to disrupt multiple carcinogenic process step. They were shown to reduce genetic damage, inhibit genetically damaged cell proliferation thanks to the induction of apoptosis and cell cycle arrest, but were also involved in malignant cells differentiation [11-12]. These anti-tumor effects may be due to the generation of Reactive Oxygen Species (ROS) that was previously reported to be one of Benzyl ITC (BITC) mechanisms of action [13-14].

In animal models, more than 20 ITCs were shown to inhibit carcinogenesis induced by several chemical carcinogens [11,15]. In human, it has been reported that those vegetables are good protective agents against several kinds of cancer: colorectal [16], lung [17] and possibly prostate cancers [18]. Among all ITCs, it was shown *in vivo* that Phenethyl Isothiocyanate (PEITC) was able to reach the highest plasma concentration after oral ingestion [19], at a micromolar dose range. Interestingly, micromolar doses of PEITC applied to animal and cell culture models were shown to prevent cancer, through several mechanisms that still need to be further investigated [10,20]. We thus wondered whether combining cisplatin with PEITC could be of potential therapeutic benefits for patients suffering from MPM, and if it could lead to less side effects and more specificity on cancer cells.

For these purposes, we focused on the anti-tumor properties of PEITC alone or in combination with cisplatin on a large collect of MPM cell lines freshly established from patients' pleural effusions in our laboratory. We demonstrated for the first time that PEITC is cytotoxic for MPM cells through ROS production. Moreover, cisplatin-PEITC combination allowed potentialization of both compounds' cytotoxic effects and prevented the emergence of resistant MPM cells. Interestingly, healthy primary mesothelial cells (PMC) were not sensitive to the combination. Finally, the presence of M2 macrophages did not change the anti-tumor properties of the combination. Our results suggest that cisplatin-PEITC combination could be of great interest for MPM treatment.

## RESULTS

### PEITC increases MPM cells cytotoxicity through ROS production

**Figure 1:**
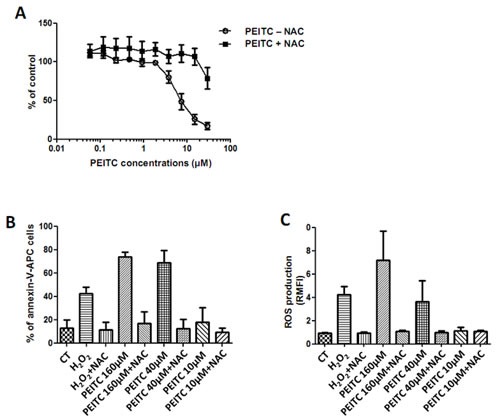
Effect of PEITC on MPM cell lines Three cell lines of MPM (Meso4, 11 and 152) were treated with increasing doses of PEITC alone or combined to NAC (5mM) for 72h. Cell viability was determined using Uptiblue reagent. Values represent the mean ± SEM of three independent measurements. B and C, MPM cell lines were treated with three doses of PEITC alone or combined to NAC (5mM) for 24h. Cell death (B) was measured by flow cytometry, after Annexin-V-APC cell staining. Cell death induction is expressed in percentage of annexin-V-APC labeled cells. ROS detection (C) was performed with flow cytometry thanks to a specific molecular probe CM-H2DCFA. Fluorescence values are expressed in Relative Mean Fluorescence Intensity (RMFI). Values represent the mean ± SEM of three independent measurements on three distinct cell lines.

PEITC was previously demonstrated to exert cytotoxic effects on tumor cells by increasing ROS intracellular level [23]. In order to evaluate the antitumor properties of PEITC, cell cytotoxic assays were conducted on three MPM cell lines: Meso4, Meso11, Meso152, treated with increasing doses of PEITC alone or in combination with NAC, a powerful antioxidant amino acid. NAC was used to highlight the implication of ROS in PEITC-induced cell death. Indeed, ROS production would be inhibited by NAC treatment. Cell cytotoxicity with PEITC treatment was increased in a dose-dependent manner, and PEITC had a similar potency on all cell lines. The IC_50_ value was 7.4 ± 0.2μM for MPM cell lines (Figure 1A). PEITC-induced cytotoxicity was inhibited by a co-treatment with NAC, suggesting the implication of ROS production in this effect.

Then, PEITC-induced ROS in MPM cells was investigated to determine whether it could be part of the mechanisms involved in cytotoxic effects on cancer cells. Hydrogen peroxide (H_2_O_2_) has very strong oxidizing properties and was used as a positive control for ROS production. Cell death induction was measured with Annexin-V cells staining (Figure 1B). ROS production was assessed by flow cytometry thanks to cells pre-incubation with the CM-H2DCFA specific fluorescent probe (Figure 1C). We observed, in a dose-dependent manner, that PEITC-induced ROS generation was consistent with PEITC-induced cell death in all tested cell lines (Figure 1B and C). In the presence of NAC, ROS generation and cell cytotoxicity were decreased, strongly suggesting the causative link between ROS generation and PEITC-induced cell death. As a control, H_2_O_2_ was shown to induce apoptosis and ROS production in MPM cells.

### Cisplatin increases MPM cells cytotoxicity partly in a ROS dependent manner

**Figure 2:**
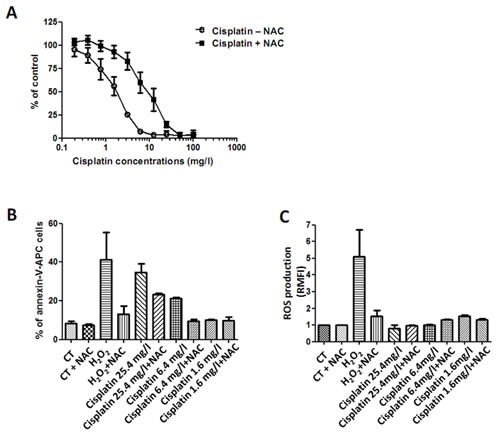
Effect of cisplatin on MPM cell lines Three cell lines of MPM were treated with increasing doses of cisplatin alone or combined to NAC (5mM) for 72h. Cell viability was determined using Uptiblue reagent. Values represent the mean ± SEM of three independent measurements. B and C, MPM cell lines were treated with three doses of cisplatin alone or combined to NAC (5mM) for 24h. Cell death (B) was measured by flow cytometry, after Annexin-V-APC cell staining. Cell death induction is expressed in percentage of annexin-V-APC labeled cells. ROS detection (C) was performed with flow cytometry thanks to a specific molecular probe CM-H2DCFA. Fluorescence values are expressed in RMFI. Values represent the mean ± SEM of three independent measurements on three distinct cell lines.

Several studies demonstrated the implication of an oxidative stress generation in cisplatin cytotoxic effects [24]. Thus, cisplatin dose-response experiments were carried out on the same three MPM cell lines previously tested. All cell lines were sensitive to cisplatin in a dose dependent manner. The IC_50_ value was 1.8 ± 0.21mg/L for MPM cell lines (Figure 2A). Treatment with NAC decreased cisplatin-induced cell cytotoxicity on all tested cell lines, arising from the possible induction of ROS by cisplatin.

To confirm the implication of an oxidative stress in the cytotoxic effect of cisplatin, we similarly investigated whether cisplatin induced-cell death is related to a ROS production and inhibited by NAC (Figures 2B and C). However, cisplatin-induced cell death did not entirely correlate with a production of ROS. Indeed, although almost 40% of cells died from a treatment with the highest concentration of cisplatin (25mg/L) (Figure 2B), yet no production of ROS was detected (Figure 2C). Cells co-treated with cisplatin (25mg/L) and NAC seemed to be more resistant to death than those untreated with NAC: almost 40% of cells died with cisplatin alone while about 23% of MPM cells died with the combination. As a control, H_2_O_2_ induced ROS production and MPM cell death. H_2_O_2_ effects were totally inhibited by a co-treatment with NAC, demonstrating its efficacy to block ROS-induced cell death.

### Cisplatin-PEITC combination potentiates their cytotoxic effect through enhanced DNA damage

**Figure 3:**
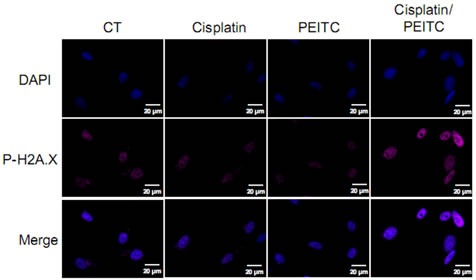
Cisplatin-PEITC combination induces the DNA recruitment of the histone H2A.X phosphorylated form in MPM cells Cells were treated with cisplatin (0.8mg/l) and PEITC (4μM) alone or in combination for 24h prior to PAF 4% fixation and immunofluorescence staining for P-H2AX. Representative images of MPM cells (Meso152) stained for P-H2A.X (pink), stained with DAPI (blue) for nuclei labeling and merge pictures.

Studies carried out on other cancer types such as non-small cell lung cancer, ovarian, testicular or cervical cancer demonstrated the implication of DNA damage in cisplatin and PEITC cytotoxic effects [25-26]. We herein evaluated on MPM cells the combination cisplatin-PEITC on DNA damage by looking at the phosphorylation on serine 139 of histone H2A.X (P-H2A.X), a specific marker for DNA damage [27] (Figure 3 and additional figure S2). No DNA damage was triggered by PEITC alone, according to P-H2A.X staining, in any cell line. While no DNA damage was observed in MPM cells after cisplatin treatment alone, combination of cisplatin with PEITC strongly enhanced P-H2A.X signal. This result suggests a potentialization of both compounds' effect on DNA damage, indeed triggering more lesions than compounds used alone. Combination of cisplatin with PEITC would thus potentiate their apoptotic effect through DNA damage.

### Cisplatin-PEITC combination enhances MPM cytotoxicity and prevents the generation of resistant cells

**Figure 4:**
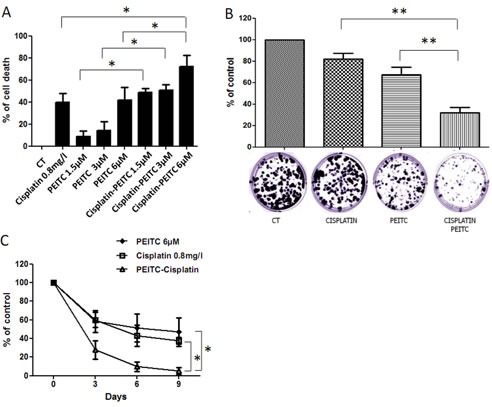
Effect of PEITC and cisplatin alone or in combination on MPM cells A, MPM cell viability assays were performed after 72h of cisplatin and/or PEITC treatments using Uptiblue cell counting reagent. Three concentrations of PEITC were used (2, 4 and 6μM), alone or in combination with cisplatin at 0.8mg/l. Values represent the mean ± SEM of three independent measurements on three distinct cell lines. B, MPM cells (Meso 4 and Meso 152) were treated with cisplatin (0.05mg/l) and PEITC (2μM) alone or in combination for 10 days. At day 10, cells were fixed, stained with Crystal Violet and coloration quantified. Histograms represent the mean ± SEM of three independent experiments performed on two MPM cell lines. C, treatments with cisplatin (0.8mg/l) and/or PEITC (6μM) were performed every three days to assess cell viability over 9 days. Viability was measured at day 3, 6 and 9 using Uptiblue cell counting reagent. Compounds concentration was adapted to 96 well plate and cell number, in order to reach at most 40% cell death after 72h of treatment. Values represent the mean ± SEM of three independent measurements on three distinct MPM cell lines. * p<0.05.

Cisplatin and PEITC cytotoxic effects after 72h of treatment were assessed using Uptiblue viability assay. Both compounds were used alone or in combination on MPM cells seeded in 96 well plates. Three concentrations of PEITC were evaluated, and cisplatin concentration (0.8mg/L) was used to trigger approximately 40% of cell death (Figure 4A). MPM cell lines died in a dose dependant manner for all PEITC concentrations. Cell death was significantly enhanced with combined compounds after 72h at all PEITC concentrations. It was the most obvious with the 6μM PEITC concentration, as there was almost 80% of cell death with the combination, when each compound alone only induced about 40% of cell death. Cisplatin-PEITC combination cytotoxic effect on MPM cells was also assessed over 10 days using a clonogenic assay (Figure 4B). Cells were seeded at a small density and treated for 10 days with PEITC and cisplatin alone or in combination. Previous dose-response experiments allowed us to choose an appropriate concentration for each compound that would not trigger more than 35% of cell death, in order to be able to observe a possible potentialization of both compounds effect with the combined treatment. After 10 days of treatment, cells were fixed and stained with crystal violet to obtain images and quantification of cell proliferation. The results shown in the figure 4B are representative of three independent experiments conducted on two MPM cell lines. Results obtained in the six experiments are provided individually as supplementary data (Figure S3). For both cell lines, PEITC and cisplatin increase cell death compared to untreated cells. PEITC is more potent than cisplatin while the combination of both compounds leads to a clear potentialization of PEITC and cisplatin effects. This result suggests that the combination of cisplatin with PEITC potentiates their cytotoxic effect on MPM cells. Furthermore, the effect of PEITC and cisplatin on MPM cells viability over time was assessed over nine days in order to establish a kinetic of both compounds used alone or in combination (Figure 4C). Viability was measured after 3, 6 and 9 days of treatment and showed that cisplatin-PEITC combination significantly potentiated both compounds' cytotoxic effects. After 3 days of combined treatment, there was about 34% viable cells while compounds alone led to approximately 60% of viable cells. At day 9, there were about 28% viable cells with cisplatin alone and 47% with PEITC. However, cisplatin-PEITC combination led to less than 5% viable cells after 9 days of treatment, thus suggesting a strong synergy when both compounds were combined. Indeed, the combination not only led to a significant increase of cell death but more importantly prevented the emergence of chemoresistance, making of this strategy a good candidate to treat reluctant cancer such as MPM.

### Cisplatin and PEITC combination is well tolerated by primary mesothelial cells

**Figure 5:**
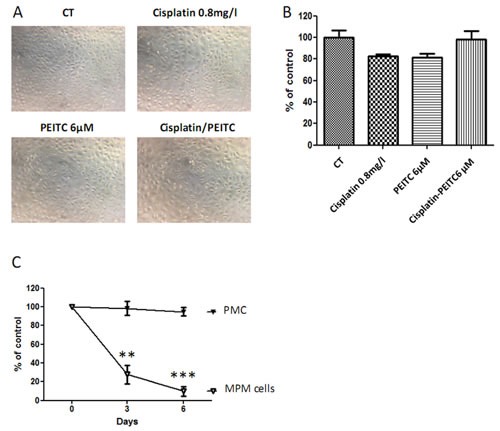
Effect of PEITC and cisplatin alone or in combination on primary mesothelial cells PMC were treated with cisplatin (0.8mg/l) and/or PEITC (6μM) for 72h. A, Microscopy pictures were taken, and B, viability assays were performed. C, Comparison of cisplatin-PEITC combination on MPM and PMC. Graphics represent the mean ± SEM of triplicates. ** p<0.01, *** p<0.001.

Aiming at evaluating the toxicity of cisplatin-PEITC combination on healthy cells, PMC were treated with cisplatin and PEITC alone or in combination for 72h. Microscopy pictures (Figure 5A) and viability assay (Figure 5B) showed that PMC were not affected by either compounds alone or combined. Moreover, a comparison between MPM and PMC clearly illustrates the safety of our strategy for healthy cells while inducing almost 100% MPM cell death (Figure 5C). This experiment suggests that the combination could be injected in pleural cavities with no side effect on cells of healthy pleura.

### Additional MPM cell lines are also sensitive to Cisplatin-PEITC combination

**Figure 6:**
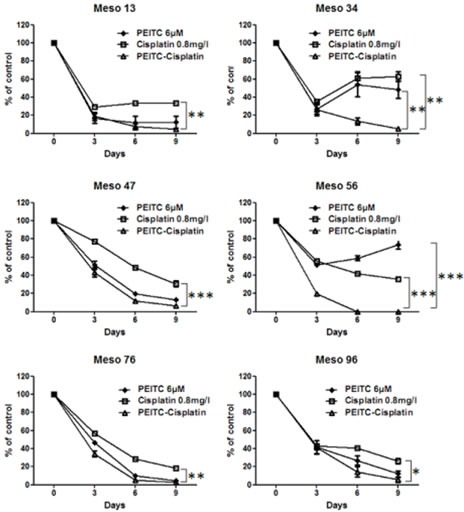
Effect of PEITC and cisplatin alone or in combination on MPM cells viability over time MPM cells treatment with cisplatin (0.8mg/l) and/or PEITC (6μM) were performed every three days to assess cell viability over 9 days. Viability was measured at day 3, 6 and 9 using Uptiblue cell counting reagent. Values represent the mean ± SEM of triplicates. * p<0.05, ** p<0.01, *** p<0.001.

The absence of cisplatin-PEITC toxicity on PMC led us to compare the sensitivity of six additional MPM cell lines from our biocollection (Figure 6). Half of MPM tested cell lines (Meso13, Meso34 and Meso56) displayed a strong resistance phenotype to cisplatin or PEITC after the second treatment repetition. Cytotoxic effect and cell death were significantly enhanced by combining both compounds, but their kinetics were variable according to each cell line. Indeed, combination best efficiency was observed at day 3 for Meso47, 56 and 76 and at day 6 for Meso13, 34 and 96. However, for all cell lines, combining both compounds significantly improved their cytotoxic effects, compared to compounds alone, indeed leading to less than 10% of viable cells after 9 days of treatment.

### Cisplatin-PEITC combination is toxic on MPM cells in the presence of M2 macrophages while remaining safe for primary mesothelial cells

**Figure 7:**
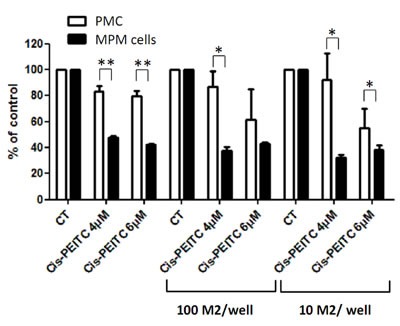
Effect of cisplatin-PEITC combination on MPM and primary mesothelial cells co-cultured with M2 macrophages MPM and PMC were co-cultured with M2 macrophages at two densities: 100 M2/well or 10 M2/well and treated with cisplatin at 0.8 mg/l in combination with PEITC at 4μM or 6μM for 72h. Viability was measured using Uptiblue cell counting reagent. Values are expressed in percentage of control and represent the mean ± SEM of experiments conducted on three MPM cell lines, or in duplicates for PMC. * p<0.05, ** p<0.01.

The tumor microenvironment is much different than the physiological one, and it is now well known that a number of factors can play an important role in the development of the tumor and in the efficiency of the treatment. As an example, M2 macrophages are cells present in the microenvironment of many cancer types, including mesothelioma, and have been described as enhancers of tumor cell proliferation [28]. Moreover, macrophages are cells that constitutively produce ROS [29], they could thus be envisioned as potentially harmful if combined to a compound also generating ROS. We thus evaluated the cytotoxic effect of cisplatin-PEITC combination on MPM and PMC in the presence of macrophages (Figure 7). A differentiation experiment was carried out from monocytes in order to obtain M2 macrophages that were phenotyped by Flow Cytometry prior to their seeding at several concentrations in 96 well plates (Figure S4), in co-culture with MPM or PMC. The following day, cells were treated with cisplatin 0.8mg/l in combination with PEITC at 4μM or 6 μM for 72h. The results show that the combination was toxic on MPM cells no matter which concentration of PEITC considered, in the presence or not of M2 macrophages. As previously observed, the combination was safe on PMC for both concentrations of PEITC. Even though the presence of macrophages seemed to sensitize PMC to the combination with the highest concentration of PEITC, the combination of cisplatin with 4μM of PEITC remained safe whereas being highly toxic on MPM cells. This experiment thus allowed us to prove that the combination cisplatin-PEITC at a dose cytotoxic for MPM cells still remains non toxic on PMC in the presence of M2 macrophages.

## DISCUSSION

Treatment of MPM represents a major public health challenge given its aggressiveness, poor prognosis after diagnosis and more importantly its lack of effective curative therapies. The first line treatment consists in a combination of cisplatin with the Pemetrexed anti-metabolite [3-4]. Although this combination showed greater results than drugs used alone, the benefit for patients remains insufficient. The investigation of new therapeutic strategies to approach this thoracic malignancy thus became a necessity.

ITCs, arising from the hydrolysis of glucosinolates contained in cruciferous vegetables, are well known for their anti-carcinogenic properties [10]. Growth of several kinds of cancer cells was shown to be inhibited by ITCs: leukemia [30-31], prostate cancer [32], breast cancer [33-34], colorectal cancer [35] etc. PEITC, a member of ITCs family, was described as an important anti-carcinogenic compound in humans [10,20]. Using MPM cell lines established in our laboratory from patients' pleural fluids, we demonstrated for the first time that these cells were sensitive to this compound. Furthermore, we showed that PEITC-induced MPM cell death by apoptosis was fully dependent on ROS production.

However, the benefit of MPM first line treatment is insufficient and needs to be improved. A previous study demonstrated an interesting potentialization of cisplatin effect when combined to PEITC in a model of lung cancer [36]. This study suggested that PEITC could be a good candidate to improve the effect of cisplatin on MPM cells.

We first characterized the effect of cisplatin on our MPM cell lines. We observed a dose dependent toxicity of cisplatin through apoptosis induction partly dependent on an oxidative stress, as demonstrated by the use of NAC. We then demonstrated that PEITC-induced cytotoxic effects were entirely mediated through oxidative stress generation in cells, while there was more than this mechanism involved in cisplatin-induced cell death mechanisms of action. Although a couple of studies recently investigated the impact of cisplatin-PEITC combination on other cancer types [37-38], our work focuses for the first time on MPM, and brings new insights for a better approach of this incurable disease. Healthy and malignant mesothelioma models had previously been investigated using similar strategies acting on the induction of an oxidative stress alone or in combination with cisplatin, confirming the relevance of these strategies [39-40]. However, we here in showed for the first time that cisplatin-PEITC combination significantly enhanced MPM cell death, potentiated DNA damage and more importantly is safe for healthy cells and prevents the emergence of resistant cells.

Cisplatin-PEITC combination showed a decrease of cell viability after 72h of treatment. Moreover, a clonogenic assay carried out over 10 days on MPM cells confirmed that the combination led to a potentialization of both compounds' cytotoxic effect. Although cisplatin and PEITC showed interesting anti-tumor activity after 72h when used alone, repeated treatments led to the apparition of resistant cells characterized by a loss of compounds' efficacy to increase cell cytotoxicity over time. Indeed, we observed, in more than half of MPM tested cell lines, a decrease of sensitivity or a resistance towards each molecule. This could explain the poor therapeutic benefit of cisplatin on MPM patients and could also suggest that PEITC used alone would have limited efficacy for its clinical use. The mechanisms of action of these two molecules were already described and mainly result in the formation of DNA damage [25-26]. Our results confirmed the adaptive capacities of tumor cells to this class of chemotherapeutic-induced lesions. However, DNA damage induced by cisplatin or PEITC is of different nature thus implying the involvement of distinct pathways of the DNA damage response (DDR) for cells to bypass those lesions. MPM patients sensitivity to cisplatin was previously correlated to mRNA expression of the excision repair cross-complementation group 1 (ERCC1) involved in the nucleotide excision repair (NER) pathway [41]. ROS production in cells acts by different mechanisms to damage DNA bases, but mainly affects guanine that gets transformed into 8-oxo-2′deoxyguanosine (8-oxoG) [42]. The main DNA repair mechanism involved to remove ROS-induced DNA lesions is the base excision repair (BER) pathway [42]. Therefore, we hypothesized that by combining cisplatin with PEITC, both BER and NER pathways would be activated. The possibility to raise resistant cells would thus be limited due to the difficulty for a cell to set up two pathways of the DDR at the same time. Our results demonstrated that all tested cell lines were sensitive to cisplatin-PEITC combination, reaching less than 5% of viable cells after three repetitions of treatment. We thus showed a correlation between cisplatin-PEITC cytotoxicity and DNA damage potentialization triggered by the combination, as illustrated by the recruitment of the phosphorylated histone H2A.X to the DNA [27]. These last results therefore demonstrated that the DDR machineries were overwhelmed, preventing the generation of resistant cells.

Interestingly, cisplatin-PEITC combination induced absolutely no toxicity on PMC while the same treatment induced MPM cell death. By performing repetitive treatments over nine days, we also demonstrated that the combination prevented the emergence of resistant tumor cells. Indeed, we confirmed that the combination was efficient on nine MPM cell lines while more than half of them became resistant to drugs used alone. These results suggest that cisplatin-PEITC combination could be an interesting strategy to treat MPM using a local administration regarding its lack of toxicity on PMC. The higher sensitivity of MPM cells to the combined treatment compared to PMC probably results from the capacity of PMC to support mild and severe oxidative stress, thanks to gluthatione redox cycle and catalase-mediated protection [43]. Usually, these anti-oxydant pathways are altered in malignant cells and associated with an increased metabolic activity. These changes lead to a higher level of ROS in malignant cells compared to healthy cells, which contribute to promote oncogenic properties such as generation of resistant cells to conventional therapies but also to sensitize cells to strong oxidative stress [42]. This combination already demonstrated its efficacy on breast cancer [37] and leukemia cells [44] while displaying no toxicity on their healthy counterpart, suggesting a good tolerability of healthy cells towards cisplatin-PEITC combination.

The tumoral microenvironment plays an important role in the development of the tumor and several actors are involved [45]. Among all cells present in the environment of the tumor, macrophages are of significant importance and have been described as enhancers of tumor cell proliferation [28]. Moreover, macrophages are cells that constitutively produce ROS [29], which could be of harm if added to a compound also generating ROS. We thus investigated the effect of cisplatin-PEITC combination on MPM and PMC in co-culture with macrophages. We showed that cisplatin-PEITC was toxic on MPM cells for both concentrations of PEITC. The presence of macrophages did not modify the sensitivity of MPM cells to the combination. Moreover, although the strongest concentration of PEITC (6μM) combined to cisplatin led to a higher sensitivity of PMC to the combination, we showed that cisplatin combined to PEITC at 4μM was toxic on MPM cells while remaining safe for PMC. The highest sensitivity of PMC to cisplatin combined to the highest concentration of PEITC thus confirms the importance of the microenvironment in the toxicity of the treatments. However, for a dose of cisplatin-PEITC highly toxic on MPM cells, we demonstrated here that this combination remains safe on PMC.

Therefore, a local administration of cisplatin-PEITC combination in pleural cavity could be a promising strategy to induce selective toxicity towards malignant cells. Altogether, cisplatin-PEITC combination would enhance cancer cell death compared to compounds used alone and prevent the emergence of cell resistance, while remaining safe for PMC.

## CONCLUSION

The combination of cisplatin with the natural compound PEITC induces a strong MPM cell death, while remaining safe for PMC, with limited emergence of cell resistance compared to drugs used alone. Therefore, this combination could represent a promising strategy for the treatment of MPM.

## METHODS

### Cell culture

The nine mesothelioma cell lines: Meso4, Meso11, Meso13, Meso34, Meso47, Meso56, Meso76, Meso96 and Meso152 were established from pleural fluids of patients [21]. These cells were characterized for the mRNA expression of the usual MPM markers by immunohistochemistry (Figure S1) and belong to a validated biocollection (Ministère de l'Enseignement Supérieur et de la Recherche n° DC-2011-1399 and Commission Nationale de l'Informatique et des Libertés (CNIL) n°: 1657097). All cell lines were maintained in RPMI medium (Invitrogen) supplemented with 2mM L-glutamine, 100IU/ml penicillin, 0.1mg/ml Streptomycin and 10% heat-inactivated fetal calf serum (Eurobio) and cultured at 37°C in a 5% CO_2_ atmosphere. Primary mesothelial cells (PMC) from peritoneal origin were purchased from Tebu-bio biosciences and cultured according to the manufacturer's recommendations.

### Drugs

PEITC, N-acetyl-cystein (NAC) and cisplatin were purchased from Sigma-Aldrich.

### Determination of cell viability

Cell viability was monitored using Uptiblue reagent (Interchim) as previously described [22]. Cells were seeded in 96-well plates at a density of 5×10^3^cells/well for MPM cells in culture medium for 24h. Then compounds were added for an additional 72h and Uptiblue reagent (5%, v/v) was then added to the culture medium for 2h at 37°C. Fluorescence was measured at 605nm after a green epi illumination excitation using a ChemiDoc™ MP imaging system (Biorad). Quantification was performed using ImageJ 1.41o software. For kinetic experiments, culture medium containing Uptiblue was replaced by medium with or without drug for 72h and the procedure for cell viability measurement was repeated twice. Results were expressed as percentage of untreated cells.

### Detection of Reactive oxygen species

Cells were seeded at a density of 1×10^6^cells/well in 12 well plates. 24h after seeding, cells were washed once with PBS and incubated for 30min at 37°C with the CM-H2DCFA probe (Life Technologies), resuspended in PBS at a final concentration of 5μM, washed once with PBS and treated with Cisplatin or PEITC alone or in combination. 24h after treatment, cells and their supernatant were harvested and analyzed by flow cytometry (FACSCalibur; Becton Dickinson). Ten thousand events were collected and analyzed with the FACS Flowjo Software (Tree Star Inc).

### Detection of apoptosis

Cells were seeded at a density of 1×10^6^cells/well in 6-well plates and treated with indicated concentrations. After 24h, floating and adherent cells were combined, labeled using Annexin V-allophycocyanin (APC) (Becton Dickinson) following the manufacturer's instructions, and analyzed by flow cytometry (FACSCalibur; Becton Dickinson). Ten thousand events were collected and analyzed with the FACS Flowjo Software (Tree Star Inc).

### Clonogenic assay

Two MPM cell lines were used to assess their effect on MPM cells proliferation: Meso4 and Meso152. This assay requires cells that grow in colonies, which was not the case of the third cell line used over this study for the other experiments (Meso11). Cells were seeded in 6-well plates at a density of 500 cells/well for Meso4 and 1000 cells/well for Meso152, according to each cell line rate of proliferation. 24h after seeding, cells were treated with PEITC alone at 2μM or in combination with cisplatin at 0.05mg/l for 10 days. At day 3, half of the medium was replaced with fresh medium, and at day 5, the totality of the medium was replaced by fresh medium until the end of the experiment. At day 10, cells were fixed with 4% paraformaldehyde in PBS and stained with 0.05% crystal violet in ethanol 70%. Cells were then imaged using a ChemiDoc™ MP imaging system (Biorad). Quantification was performed using ImageJ 1.41o software.

### Co-culture assay

MPM and PMC were co-cultured with M2 macrophages and treated with cisplatin and PEITC alone or in combination for 72h. Three MPM cell lines were used: Meso4, 11 and 152, seeded in a 96 well plate at a density of 5.10^3^ cells/well. According to cell rate proliferation, primary mesothelial cells were seeded at a density of 2.10^4^ cells/well in order to create a cell layer similar to the physiological conditions. M2 macrophages were obtained from monocytes that were differentiated with a treatment with M-CSF (50ng/ml). As a control, a fraction of monocytes was also treated with GM-CSF (20ng/ml) to obtain M1 macrophages. After 5 days, both M-CSF and GM-CSF-treated monocytes were harvested and characterized by flow cytometry using CD14-FITC and CD163-APC staining. M2 macrophages were seeded in co-culture with MPM or PMC at two concentrations: 100 and 10 macrophages per well. 24h after seeding, cells were treated with cisplatin at 0.8mg/l in combination with PEITC at 4μM or 6μM for 72h. Cell viability was then measured using Uptiblue counting reagent as described above. Fluorescence was measured at 605nm after a green epi illumination excitation using a ChemiDoc™ MP imaging system (Biorad). Quantification was performed using ImageJ 1.41o software.

### Microscopic experiments

Cells were seeded in culture medium on glass coverslips at a density of 5×10^4^cells/well in 12 well plates. 24h later, drugs alone or in combination were added for additional 24h. Cells were washed twice with PBS and fixed with 4% paraformaldehyde in PBS, 15min at room temperature. Cells were permeabilized with 0.05% Triton X-100 (Merck) /0.05% Tween-20 (Sigma-Aldrich) in PBS (5min) and incubated with anti-γH2A.X (phospho S139) antibody at 1μg/ml (Abcam) in PBS/1% BSA for 1h. Cells were washed twice with PBS and incubated with a DyLight^tm^ 633 conjugated secondary antibody (Thermo Scientific) for 1h. After an additional PBS wash, cell nuclei were stained with 1μg/ml Hoechst (Sigma-Aldrich) (5min). Coverslips were mounted in ProLong^®^ Gold (Molecular Probes) and fluorescence was visualized using the Axiovert200M microscopy system (Zeiss, Le Pecq, France) with ApoTome module (X63 and numerial aperture 1.4).

### Statistical analysis

Statistical analyses were performed using GraphPad prism, (Prism 5, Windows). Data are expressed as the means ± S.E.M. of at least three experiments. Statistical comparisons were made using the nonparametric Mann-Whitney test.

## SUPPLEMENTARY MATERIAL FIGURES


