# Supplementary material for: Cisplatin in combination with Phenethyl Isothiocyanate (PEITC), a potential new therapeutic strategy for malignant pleural mesothelioma
Source: Oncotarget. 2014 Oct 18;5(22):11641–52. doi: 10.18632/oncotarget.2604 (PMC4294387; doi:10.18632/oncotarget.2604)
Supplement: Supplementary file 1 [file oncotarget-05-11641-s001.pdf]

Cisplatin in combination with Phenethyl Isothiocyanate (PEITC), a potential new therapeutic strategy for malignant pleural mesothelioma

Supplementary Material

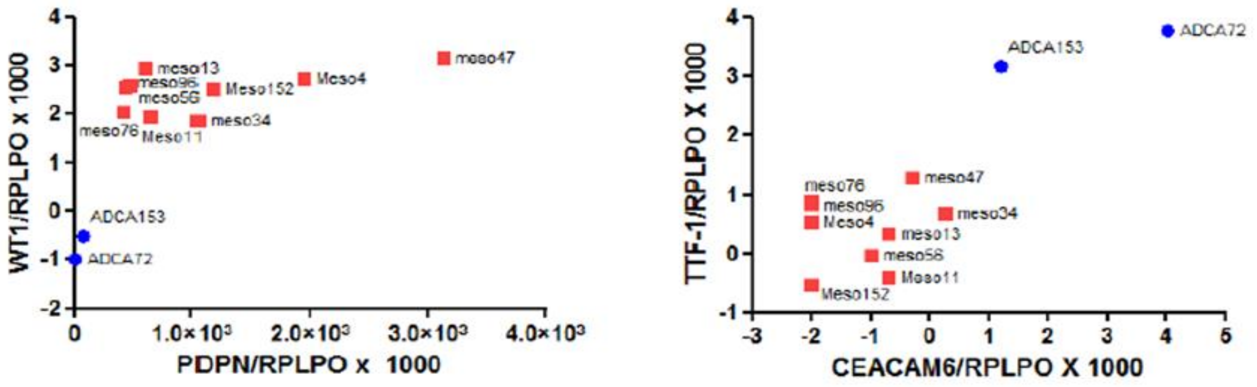

Figure S1 : Characterisation of MPM and lung ADCA cell lines established from patients' pleural effusion. Expression of mRNA of usual immunohistochemical markers used to diagnose MPM were measured using real-time PCR. A, mRNA expression of MPM markers Wilm's Tumor antigen (WT1) and podoplanin (PDPN). B, mRNA expression of lung ADCA markers Thyroid transcription factor-1 (TTF-1) and Carcinoembryonic antigen-related cell adhesion molecule-6 (CEACAM6).

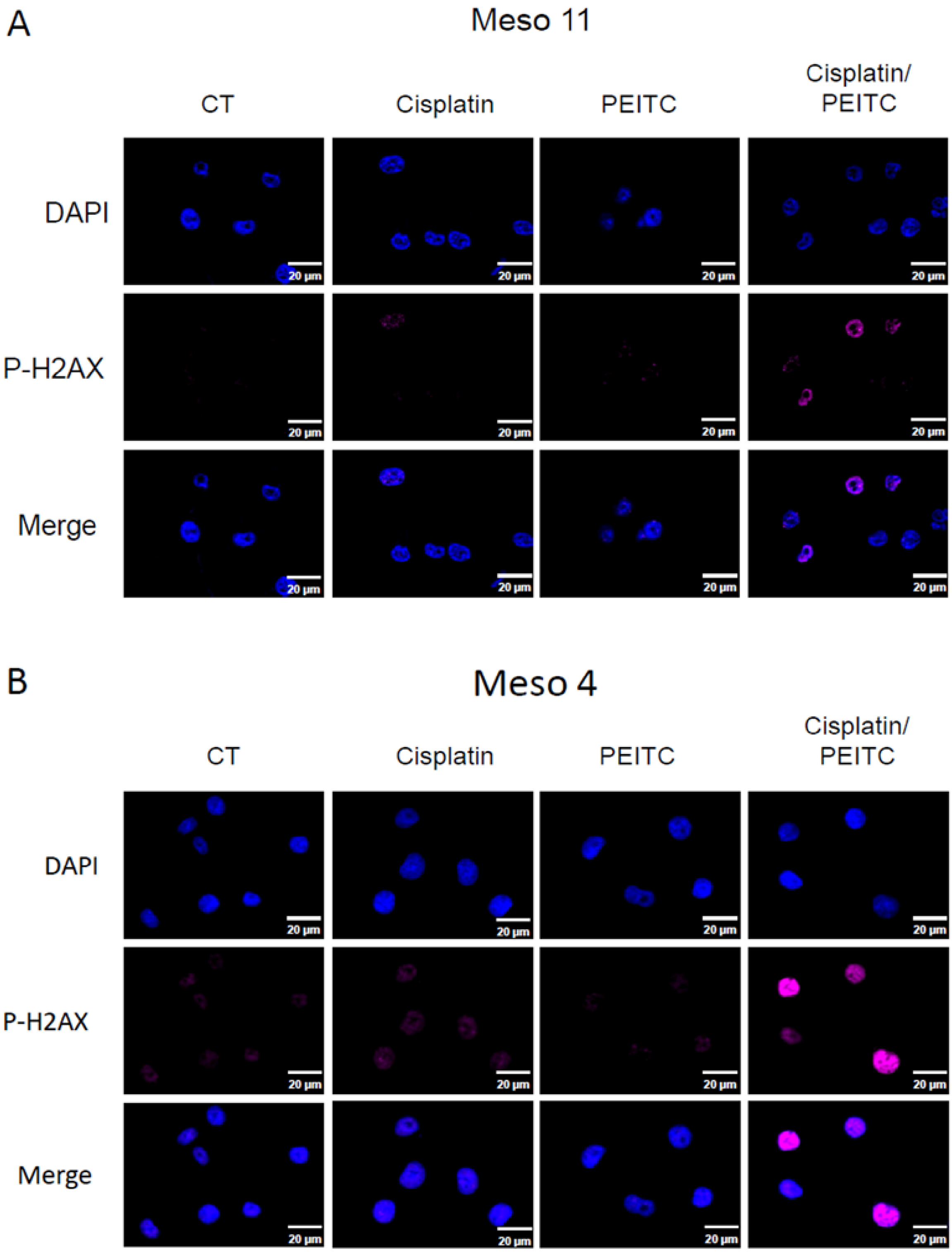

Figure S2: Effect of PEITC and Cisplatin alone or in combination on the DNA recruitment of the phosphorylated form of histone H2A.X in MPM cells.
Cells were treated with cisplatin (0.8mg/l) and PEITC (4µM) alone or in combination for 24h prior to PAF 4% fixation and immunofluorescence staining for P-H2AX. Representative images of MPM cells (Meso11 and Meso4) stained for P-H2A.X (pink), stained with DAPI (Blue) for nuclei labeling and merge pictures.

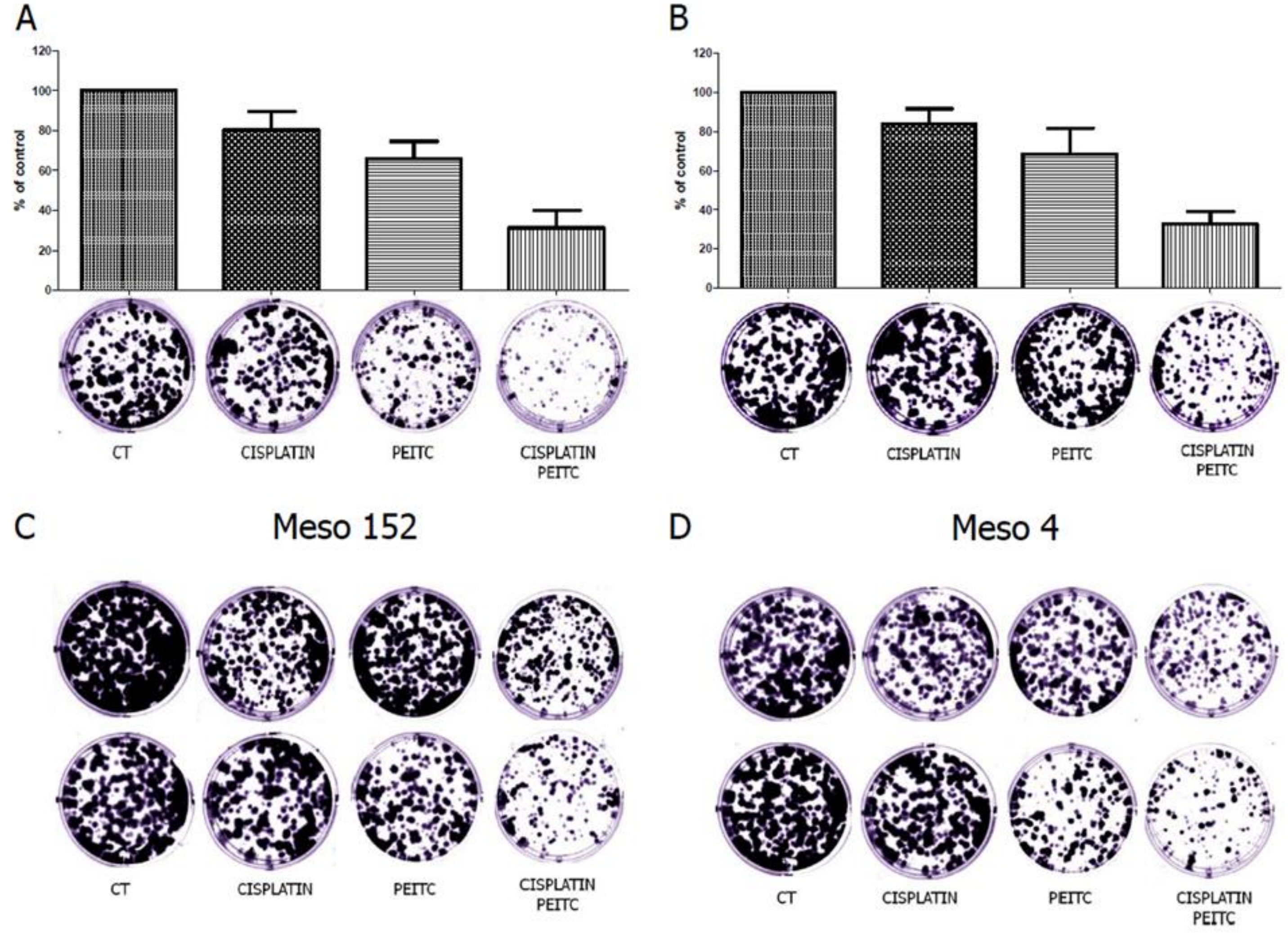

Figure S3: Effect of PEITC and cisplatin alone or in combination on MPM cells clonogenic properties.

MPM cells were treated with cisplatin (0.05mg/l) and PEITC (2μM) alone or in combination for 10 days. At day 10, cells were fixed and stained with Crystal Violet. Cell wells were imaged and coloration quantified. A and B show the values of the mean ± SEM of coloration quantification of three independent experiments for both cell lines. C and D show the images obtained for both cell lines in the two other independent experiments.

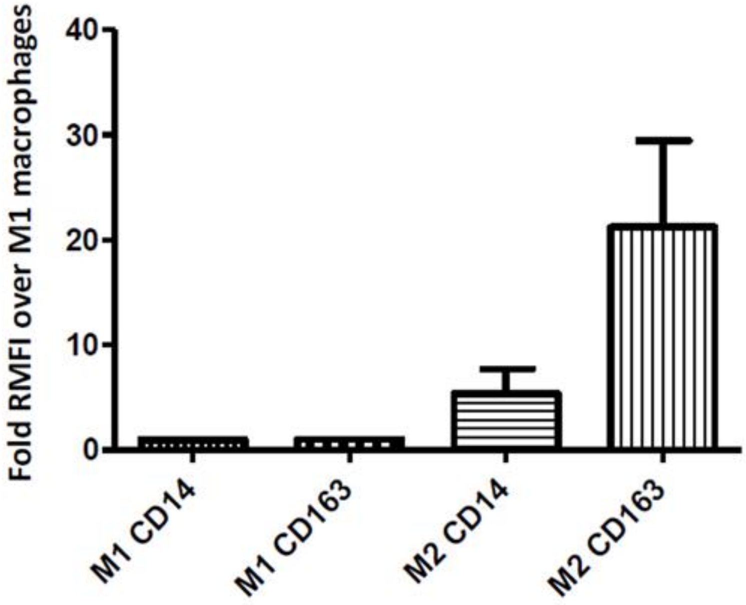

Figure S4: Characterization of M2 macrophages.

M2 macrophages were obtained from monocytes that were differentiated with a treatment with M-CSF (50ng/ml). As a control, a fraction of monocytes were also treated with GM-CSF (20ng/ml) to obtain M1 macrophages. After 5 days, both M-CSF and GM-CSF treated monocytes were harvested and characterized by flow cytometry using CD14-FITC and CD163-APC staining. Values represent the mean ± SEM of three independent monocytes' differentiation experiments.
